# Exploring the role of mTOR pathway in aging and age-related disorders

**DOI:** 10.17179/excli2025-8384

**Published:** 2025-08-04

**Authors:** Komal Raghuvanshi, Disha Raghuvanshi, Dinesh Kumar, Eugenie Nepovimova, Marian Valko, Kamil Kuca, Rachna Verma

**Affiliations:** 1 School of Biological and Environmental Sciences, Shoolini University of Biotechnology and Management Sciences, Solan 173229, Himachal Pradesh, India; 2 School of Bioengineering and Food Technology, Shoolini University of Biotechnology and Management Sciences, Solan 173229, Himachal Pradesh, India; 3 Center of Advanced Innovation Technologies, VSB- Technical University of Ostrava, 70800, Ostrava-Poruba, Czech Republic; 4 Department of Chemistry, Faculty of Sciences, University of Hradec Kralove, 50005 Hradec Kralove, Czech Republic; 5 Faculty of Chemical and Food Technology, Slovak University of Technology, 812 37, Bratislava, Slovakia; 6 Centre for Basic and Applied Research, Faculty of Informatics and Management, University of Hradec Kralove, 50005 Hradec Kralove, Czech Republic; 7 Biomedical Research Center, University Hospital Hradec Kralove, 50005, Hradec Kralove, Czech Republic

**Keywords:** aging, mTOR, mTOR inhibitors, rapamycin, anti-aging interventions

## Abstract

Aging is a highly intricate biochemical process. There is strong evidence suggesting that organismal aging, age-dependent diseases, and cellular senescence are related to the mammalian target of rapamycin (mTOR) signaling pathway. The signaling pathway of mTOR has become a prominent regulatory hub, managing crucial cellular activities that significantly affect lifespan and longevity. The mTOR is involved in controlling cell growth and metabolism in response to both internal and external energy signals as well as growth factors. The interaction between mTOR and cellular homeostasis is crucial in the aging process. This extensive review summarizes the most recent findings on mTOR inhibitors in the context of aging, highlighting their complex interactions with cellular systems, effect on longevity, and potential as therapeutic approaches for age-related diseases. Rapamycin and rapalogs (analogs of rapamycin), which have been proven to be effective mTOR inhibitors, have the ability to reduce the aging process in several model species while also enhancing metabolic health and stress responses. Despite cellular factors, mTOR inhibitors have revealed a potential path for therapeutics in age-related illnesses. These results suggest mTOR inhibitors as potential therapies to address the complex aspects of age-related diseases. However, obstacles stand in the way of clinical translation. Further research is required to improve dosing protocols, reduce potential side effects, and target mTOR inhibitors precisely at specific tissues. In summary, the mTOR signaling pathway is an important node in the intricate web of aging and its associated disorders.

## 1. Introduction

**Figure 1:**
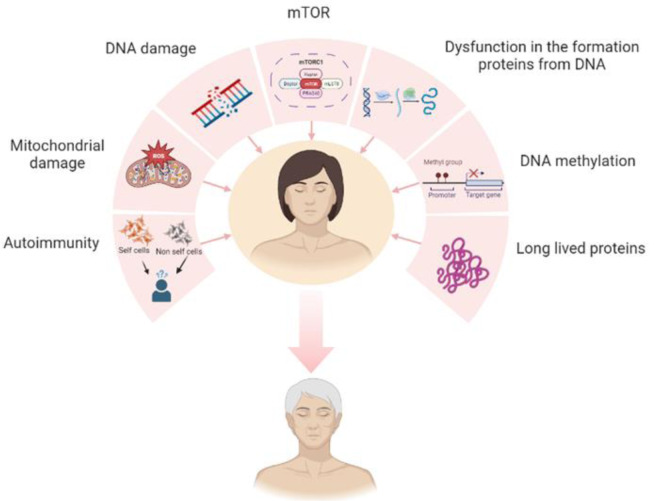
Figure 1. Major causes of aging

Aging is defined as the progressive loss of vital functions of cells influenced by time that affects every living organism (Diamanti-Kandarakis et al., 2017[28]). It is an inevitable event and is an intricate biological process (Getoff, 2007[41], Ho et al., 2010[47]). In terms of appearance wrinkles, sagging skin and other signs of aging might be visible on the skin (Masaki, 2010[83]). In the 1950s, the cause of aging was unknown, making it an 'unsolved biological mystery' (Holliday, 2006[49]). Following an extensive study on the subject, it is now commonly understood that aging is a multifaceted occurrence caused by the cumulative impacts of dietary variables, genetic variation, lifestyle choices, and environmental risk factors (Ho et al., 2010[47]). One of the early theories on aging was that chromosomal and gene mutations build up over time and eventually cause aging. We now understand that chromosomal alterations and somatic variations both increase with age (Morley et al., 1982[88]; Holliday, 2000[51]). Another widely accepted notion is that reactive oxygen species (ROS) disrupt deoxyribonucleic acid (DNA), proteins, membranes, and organelles (Cutler and Rodriguez, 2003[23]). Mitochondrial DNA is a feasible target for ROS. Advanced molecular techniques have identified numerous irregularities in mitochondrial DNA within the cells of elderly individuals. Therefore, the suggestion that these mitochondrial abnormalities could be a primary factor driving the aging process is not surprising (Linnane et al., 1989[76]; Holliday, 2004[50]). Numerous studies have shown that long-lived proteins experience a wide range of aberrant chemical changes in their amino acids, such as improper phosphorylation and methylation, racemization, glycation, deamidation, oxidation, and incomplete breakdown of the protein structure itself. Certain modified proteins, like advanced glycation end products (AGEs), assemble into clumps within secondary lysosomes and are difficult to break down. Unsurprisingly, it has frequently been argued that these alterations are a major cause of aging (Rattan, 1996[99], Rothstein, 2012[102]). Although systems exist to assure the precision of DNA to RNA (Ribonucleic acid) and RNA to protein synthesis, if they fail due to any of a number of factors, then the cells are sent on a downward trajectory that cannot be stopped, which is also believed to be a contributing factor to aging (Holliday, 2004[50]). Substantial studies have demonstrated that the body's defense system becomes less effective as we age, leading to the theory that the systems that typically distinguish self-antigens from non-self-antigens weaken with time. This results in autoimmunity, a process that gradually damages healthy cells or tissues and may negatively impact a number of processes, and it is believed to be a key contributor to aging and age-related illnesses as shown in Figure 1(Fig. 1) (Mishra and Kammer, 1998[86], Stacy et al., 2002[109]). It has recently been found that differentiated cells are kept intact by epigenetic processes. According to the ''dysdifferentiation'' notion, alterations in the signals that govern the epigenotype, such as DNA methylation, may lose specificity with age (Holliday, 2004[51], Wareham et al., 1987[119]). The mTOR pathway is involved in aging in a variety of model organisms, and studies have conclusively demonstrated that increased mTOR activity is a characteristic of aging (Xu et al., 2013[122]). There is strong evidence that the aging mechanism of numerous species including mammals, worms, yeast, and flies is significantly influenced by mTOR signaling. In eukaryotic cells, mTOR controls a wide range of vital processes, including autophagy and protein synthesis. The development of diabetes, cancer, and aging have all been related to aberrant mTOR signaling (Liu et al., 2019[77]) Yeast, specifically *Saccharomyces cerevisiae*, was the source of the initial evidence linking aging and mTOR signaling. Studies on yeast have shown that altering the mTOR pathway can affect the aging process.

Researchers found that yeast's chronological lifetime may be increased by deleting the gene Sch9, which is a homolog of the mTORC1 (mammalian target of rapamycin complex 1) substrate Ribosomal S6 kinase (S6K). Later research on *Caenorhabditis elegans* demonstrated that mTORC1 activity may shorten multicellular organisms' lifespans. Longevity was improved in this model organism by RNA interference (RNAi)-mediated reduction of key mTORC1 components such as let-363 or daf-15 (homologs of mTOR and RAPTOR, respectively). Research on *Drosophila melanogaster* has provided additional evidence in favor of the function of mTOR in controlling longevity. Research has shown that modifying the mTOR pathway can change the lifespan of *Drosophila melanogaster* (Johnson et al., 2015[61]). 

Anti-aging has always piqued everyone's curiosity (Ho et al., 2010[47]). We are aware that the aging process cannot be stopped (Hayflick, 2004[45]). Scientists have made significant advances in understanding the biology of aging in the last ten years. The aging process was once believed to be unavoidable and unchangeable, but it can now be influenced by environmental, genetic, and pharmaceutical factors (Kaeberlein, 2013[64]). Anti-aging treatment now aims to increase health span rather than longevity (Ho et al., 2010[47]). Antiaging therapies today include topical medications such as lotions and serums, microdermabrasion treatment, botox injections, and creams. The end result of these procedures is to reverse the dermal and epidermal aging indications in order to achieve youthful, clear, transparent, and resilient skin (Fibrich and Lall, 2018[37]). The ability of mTOR inhibitors to slow down the aging process has attracted a lot of attention in recent years. Rapamycin and its derivatives, which hinder the activity of mTOR, precisely target and restrict mTOR activity, resulting in the inhibition of mTOR-mediated signaling pathways. These inhibitors show the potential for prolonging life, improving health, and reducing age-related illnesses by altering the signaling of mTOR (Arriola Apelo et al., 2016[4], Blagosklonny, 2013[14]). There are several different ways in which mTOR inhibitors affect aging. Inhibiting mTOR impacts essential cellular processes associated with aging which include, autophagy, metabolism, cellular senescence, and protein synthesis. Additionally, mTOR signaling couples with other important networks linked to aging, such as the AMP-activated protein kinase (AMPK) pathway and the insulin/IGF-1 signaling system, impacting the aging mechanism (Johnson et al., 2015[61]). Even while mTOR inhibitors have the potential to support healthy aging, there are still a number of difficulties and factors to take into account. The transition of mTOR inhibitor medicines from laboratory experiments to human clinical trials requires meticulous consideration of safety, dose, and possible adverse effects. Additionally, there is still an ongoing study on mTOR inhibitors to treat different age-related disorders in different populations (Johnson and Kaeberlein, 2016[59]).

This review aims to present a comprehensive analysis of the role of mTOR signaling in aging as well as an overview of the current state of the research regarding the potential use of mTOR inhibitors as anti-aging therapies. We will look into how mTOR inhibitors affect cellular and organismal aging processes such as stem cell function, autophagy, protein synthesis, autophagy, and cellular senescence. In addition, we'll talk about the challenges and risks that come with putting these discoveries to use in clinical trials, such as the best dose, safety concerns, and feasible treatment. This review aims to contribute information on the potential of mTOR inhibition as an intervention for enhancing healthy aging and increasing overall health span by emphasizing the mechanisms of mTOR inhibitors. Ultimately, a deeper knowledge of the mTOR pathway and its regulation by pharmacological treatments may clear the path for the development of potent anti-aging therapeutics capable of improving the health and quality of human life in the aging population.

## 2. mTOR Signaling and Aging

Understanding mTOR signaling might give a complete approach to combating both aging and age-related disorders. Due to its ability to alter a wide range of activities that are important in aging, the mTOR signaling mechanism is an intriguing option for investigating both aging and the diseases associated with it (Perluigi et al., 2015[95]). For instance, growth, proliferation, and cellular metabolism are processes that are largely regulated by mTOR. Therefore, it has been determined that mTOR plays a part in aging (Zaytseva et al., 2012[124]).

### 2.1. Background of mTOR 

#### 2.1.1. Discovery and identification of mTOR 

Serine/threonine kinase mTOR, which is highly conserved in eukaryotes, is essential in recognizing and reacting to signals related to nutrition availability and growth (Johnson et al., 2015[61]). From its inhibitory compound, rapamycin originates the term "TOR" (target of rapamycin) (Weichhart, 2018[120]). In early 1970, Dr. Suren Sehgal, while working at Ayerst Laboratories, made the discovery from samples of soil taken from the Rapa Nui, an island in Polynesia had a unique antifungal characteristic due to the presence of *Streptomyces hygroscopicus. *Thus, the chemical identified from *Streptomyces hygroscopicus* was termed rapamycin (Vézina et al., 1975[117]). In 1975, extensive research was conducted on Rapamycin (also termed sirolimus) used primarily as an immunosuppressant, prior to the complete understanding of its actions. In 1984, Researchers discovered that rapamycin's suppressive effect on mTOR has impacted other than immunosuppression. The drug's potential anti-cancer capabilities were investigated, as mTOR is important in cell growth and proliferation. In 1991, target of rapamycin 1 (TOR1) and target of rapamycin 2 (TOR2) genes were first identified as hereditary controllers responsible for regulating the growth-suppressing impacts of rapamycin in the yeast *Saccharomyces cerevisiae.* Soon later, mammalian cells' mTOR protein was isolated, and it was shown that it was the rapamycin drug's actual physical target. After that, it was approved for use in post-transplantation treatment in 1999. During that period, the term "rapamycin" has been used to refer to rapamycin as well as its many derivatives, including everolimus, temsirolimus, ridaforolimus, and zotarolimus (Johnson et al., 2013[60], Sabatini et al., 1994[103]). Rapamycin has received interest recently due to its ability to increase longevity and promote better aging. Rapamycin therapy has been found in animal studies to enhance longevity and postpone the initiation of diseases associated with aging, generating interest in its potential use in aging-related therapies.

#### 2.1.2. mTOR complexes

**Figure 2:**
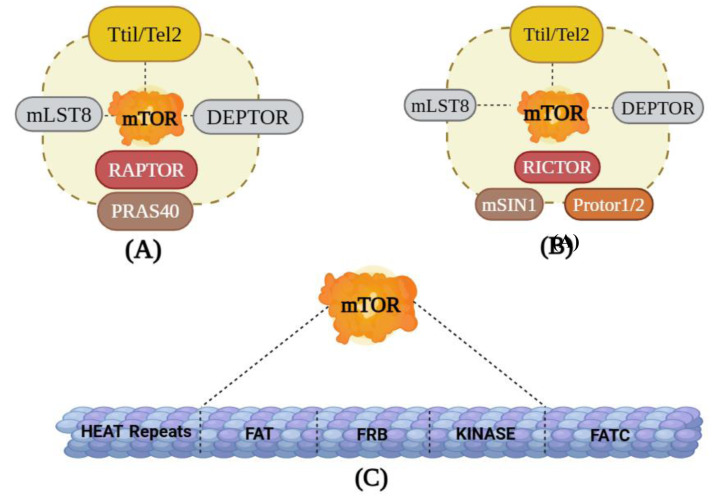
Figure 2. Structures of mTOR: (A) mTORC1: This contains six distinct protein elements like mTOR, DEPTOR (DEP domain containing mTOR interacting protein) Tti1/Tel2 complex (Catalytic subunit), RAPTOR (mTOR regulatory protein), PRAS40 (proline-rich Akt substrate), and the mLST8 (mammalian lethal with Sec13 protein 8); (B) mTORC2: mTORC2 is made up of seven protein groups that include mTOR, mLST8, the Tti1/Tel2 complex, DEPTOR, Protor1/2, mSin1 (mammalian stress-activated protein kinase-interacting protein 1), and RICTOR (rapamycin-insensitive companion of mTOR; (C) mTOR: This contains five distinct protein domains that makeup mTOR including Huntingtin-Elongation factor 3-regulatory subunit A of PP2A-TOR1 repeats (HEAT repeats), the FAT-carboxy terminal domain (FAT domain), FKBP12-rapamycin binding domain (FRB domain), FRAP-ATM-TTRAP domain (FATC domain), and Kinase.

A serine/threonine protein kinase, mTOR belongs to the phosphatidylinositol 3-kinase-related kinase protein (PIKK) family (Jung et al., 2010[62]). protein synthesis, mortality, and proliferation are just a few of the processes that mTOR regulates in cells. It is a protein that is widely distributed throughout cells and is mostly found in the cytoplasm of the cell, where it aids in synthesis processes (Abelaira et al., 2014[1]). It is a functioning enzyme that is a part of two separate complexes called mTORC1 and mTORC2, each of which has unique protein partners and substrates. mTORC1 contains six distinct protein elements like mTOR, Tti1/Tel2 complex (Catalytic subunit), DEP domain mTOR interacting protein (DEPTOR), Regulatory-associated protein of mTOR (RAPTOR), proline-rich Akt substrate of 40kDa (PRAS40), and the mammalian lethal with Sec13 protein 8 (mLST8) as shown in Figure 2(Fig. 2). The five distinct protein domains that make mTOR include Huntingtin-Elongation factor 3-regulatory subunit A of PP2A-TOR1 repeats (HEAT repeats), the FAT-carboxy terminal domain (FAT domain), FKBP12-rapamycin binding domain (FRB domain), FRAP-ATM-TTRAP domain (FATC domain) and kinase. RAPTOR is a structural protein that controls intricate assembly and substrate identification. It is a protein, that has a molecular weight of 150 kDa, attaches itself to proteins that have the TOR signaling, and transfers them to the mTOR catalytic domain. RAPTOR binds to the FRB domain in competition with rapamycin and coordinates the formation of the mTORC1 complex, leading to the activation of its catalytic function (Hoeffer and Klann, 2010[48]). Additionally, RAPTOR is an essential amino acid sensor that regulates mTORC1's subcellular location (Sancak et al., 2008[105]). Although mLST8 connects with the catalytic component of mTORC1 and may maintain the kinase activation loop, genetics research shows that mLST8 is not required for the vital functions of mTORC1. Negative regulators of mTORC1 include PRAS40 and DEPTOR, by preventing mTORC1 from accessing its substrates, PRAS40 controls the relationship between mTOR and RAPTOR and negatively controls mTOR signaling. In response to mTORC1 activation, PRAS40, and DEPTOR function can be directly phosphorylated and decreased (Dunlop and Tee, 2009[31]). mTORC2 is made up of seven protein groups that include mTOR, mLST8, the Tti1/Tel2 complex, and DEPTOR. mTORC2 does not contain RAPTOR but contains mammalian stress-activated protein kinase-interacting protein 1 (mSin) which has an unknown function but is required because Sin1 (stress-activated protein kinase-interacting protein 1) deletion is embryonically lethal (Laplante and Sabatini, 2012[73]), Protor1/2, and rapamycin-insensitive companion of mTOR (RICTOR), a different protein with similar functions as a RAPTOR. Rapamycin hinders mTORC1 by preventing its connection with RAPTOR when RAPTOR binds to the FK506-binding protein 12 (FKBP12). On the other hand, short-term rapamycin treatment does not affect mTORC2. Remarkably, prolonged rapamycin exposure impairs mTORC2 signaling even though the complexes involving rapamycin and FKBP12 do not attach to or obstruct mTORC2. Increasing mTORC2 levels causes Akt signaling to be hampered because rapamycin-bound mTOR is unable to create new mTORC2 complexes or alter the mTORC1/C2 equilibrium (Liu et al., 2019[77]). 

#### 2.1.3. Signaling of mTOR

##### Signaling of mTORC1

Lysosomes are an organelle where mTORC1 is activated (Benjamin and Hall, 2013[11]). When growth factors like insulin interact, a series of phosphorylation events occur, triggering a cascade that activates several proteins, including extracellular-signal-regulated kinase 1/2 (Erk1/2), phosphoinositide-3-kinase-related family (PI3K), p90 ribosomal S6 kinase 1 (RSK1), and Protein kinase B (PKB, also termed as AKT kinase). As a result, Erk1/2 and RSK1 phosphorylate the TSC2-TSC1 (tuberous sclerosis complex 1-tuberous sclerosis complex 2) protein complex, preventing it from performing its regulatory function. This action reduces TSC1/2's suppressive effect on mTORC1, due to which mTORC1 activates (de Cavanagh et al., 2015[25]). Additionally, Akt directly stimulates mTORC1 by phosphorylating PRAS40, that hypothesized to cause PRAS40 to separate from mTORC1 and reduce its inhibitory effect on mTORC1 activity (Laplante and Sabatini, 2009[72], Laplante and Sabatini, 2012[73]).

Additionally, low oxygen levels affect mTORC1 function by triggering hypoxia-related transcriptional activity in DNA damage-inducible transcript 4 (DDIT4), also termed REDD1. As a result, TSC2 can no longer effectively obstruct mTORC1 signaling by interacting less favorably with its inhibitory proteins (Weichhart et al., 2015[121]). 

Intracellular amino acids and mTORC1 interaction are complex and not fully understood (Efeyan et al., 2015[32]). Amino acid-induced signaling causes mTORC1 to move from the cytosol to the lysosome surface, where Rheb activation takes place. When Rheb is in its GTP-bound form at the lysosome, it increases mTORC1 activity (Inoki et al., 2003[55]). Rag GTPases, namely GDP-bound RagC/D and GTP-bound RagA/B, are required for the amino acid-induced translocation of mTORC1 from the cytosol to the lysosomes. Rheb activates mTORC1 by increasing its kinase activity, which eventually leads to the phosphorylation of downstream targets and promotes protein synthesis (Takahara et al., 2020[112]).

**Figure 3:**
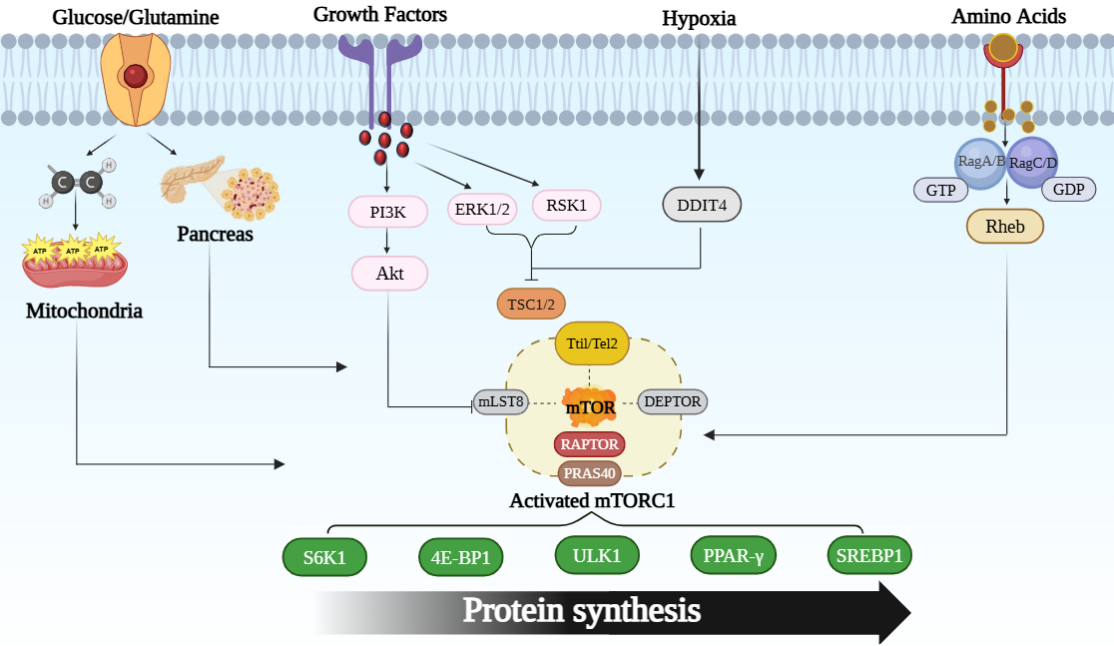
Figure 3. Diagrammatic depiction of the mTORC1 signaling route: Growth factors, amino acids, glucose, and glutamine from the extracellular environment all interact with cell membrane through specific receptors present on them, these factors directly or indirectly activate the mTORC1. When glucose levels are abundant, it leads rise in the production of ATP (adenosine triphosphate). Increased ATP levels inform mTORC1 that enough energy is available for protein synthesis and cell growth. Glucose indirectly influences mTORC1 through insulin. When blood glucose levels rise, the pancreas releases insulin, triggering the PI3K-Akt pathway, which then influences mTORC1.When growth factors adhere to their receptors, a chain of signaling events is initiated that activates ERK1/2 (Extracellular Signal-Regulated Kinase 1/2), RSK1 (p90 Ribosomal S6 Kinase 1), and PI3K (phosphoinositide 3-kinase) triggering a signaling cascade. This process results in the activation of Akt (Protein Kinase B). Akt phosphorylates and blocks PRAS40 (a 40 kDa proline-rich Akt substrate). As a result of this action, PRAS40 detaches from mTORC1 and reduces its ability to inhibit mTORC1 activity. ERK1/2 and RSK1-mediated phosphorylation of the tuberous sclerosis complex (TSC2) can inhibit the TSC1-TSC2 complex's suppressive effect on mTORC1, thereby promoting mTORC1 activation. DDIT4 released or activated in response to hypoxia. DDIT4 (DNA damage-inducible transcript 4) inhibit TSC1-TSC2 complexes, resulting in the activation of mTORC1. When amino acids enter the cell, they activate heterodimeric complexes composed of RagA/B paired with GTP and RagC/D paired with GDP. On the lysosomal surface, Rag GTPases interact with another protein called Rheb (Ras homolog enriched in brain). this process results in the activation of Rheb, which subsequently activates mTORC1. Once activated, mTORC1 triggers key signaling molecules like S6K1 (Ribosomal S6 kinase 1), 4E-BP1 (Eukaryotic translation initiation factor 4E (eIF4E)-binding protein 1), ULK1 (Unc-51-like autophagy-activating kinases 1), PPAR-γ (Peroxisome proliferator-activated receptor gamma), and SREBP-1 (Sterol Regulatory Element-binding Protein-1)

Both glucose and glutamine help to activate mTORC1 by supplying carbon compounds to power the mitochondrial tricarboxylic acid (TCA) cycle for ATP production as shown in Figure 3(Fig. 3). When glucose levels are abundant, it leads to a rise in the production of adenosine triphosphate (ATP). Elevated ATP levels signal to mTORC1 that there is sufficient energy available for protein synthesis and growth of cells. In addition to ATP, glucose also indirectly influences mTORC1 through the insulin signaling pathway. The release of insulin from the pancreas can be triggered by elevated blood glucose levels. Insulin then triggers the PI3K-Akt pathway, Akt directly stimulates mTORC1 by phosphorylating PRAS40, that hypothesized to cause PRAS40 to separate from mTORC1 and reduce its inhibitory effect on mTORC1 activity. 

Increased processes like mRNA synthesis, translation initiation and translation elongation, and the synthesis of ribosomal proteins result from the activation of S6K1 by mTORC1. This activation also causes a cascade of effects on numerous downstream proteins. Phosphorylation of 4E-BP1 inhibits its interaction with the eukaryotic translation initiation factor 4E (eIF4E), enabling eIF4E to facilitate cap-structured translation. Additionally, when growth factors and nutrients are present mTOR suppresses autophagy by phosphorylating Ser 757, which inhibits unc-51-like kinase 1 (ULK1). On the other hand, in times of nutrient scarcity, AMPK phosphorylates Ser 317 and Ser 777 directly, activating ULK1 and subsequently promoting the start of autophagy (Kim et al., 2011[67]). The activation of mTORC1 causes an increase in the production of new lipids. This activation stimulates the transcription of genes encoding proteins involved in maintaining stable lipid and cholesterol levels, such as peroxisome proliferator-activated receptor-gamma (PPAR-gamma) and sterol regulatory element binding protein 1 (SREBP1) (de Cavanagh et al., 2015[25]).

##### Signaling of mTORC2

**Figure 4:**
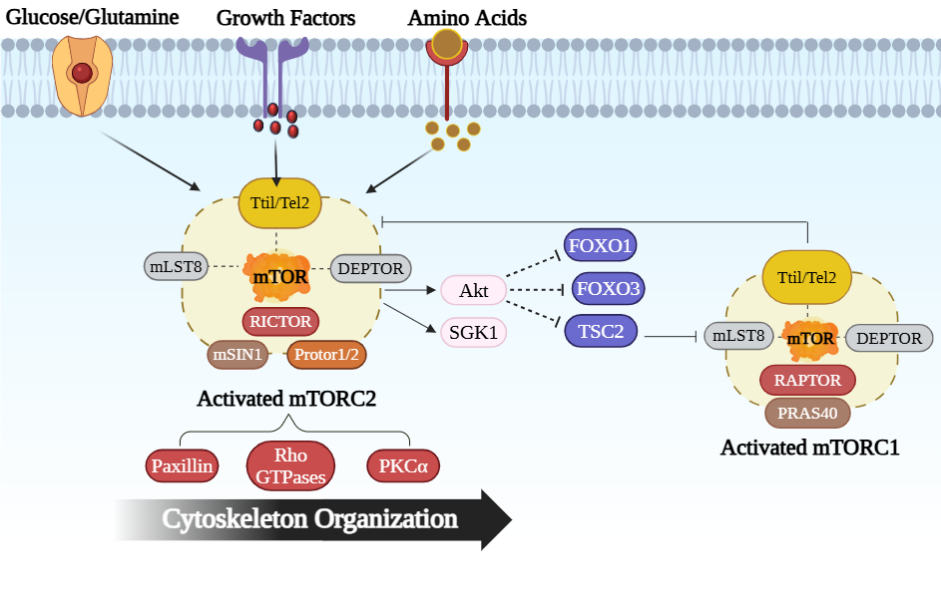
Figure 4. Diagrammatic depiction of the mTORC2 signaling route: mTORC2 is primarily activated upon receiving signals from Glucose and glutamine, growth factors, and amino acids signaling. One of the primary targets of mTORC2 is PKB/Akt (Protein kinase B) and SGK1 (serum- and glucocorticoid-induced protein kinase 1). Which then phosphorylates the transcription factors forkhead box O1 (FOXO1) and FOXO3 and TSC2 (tuberous sclerosis complex 2) restricting their activity which causes mTORC1 to be activated, which subsequently leads to restriction of mTORC2. mTORC2 also activates Paxillin, Rho GTPaese, and PKC-α (Protein kinase C alpha) which regulate the cytoskeleton organization.

When compared to mTORC1, little is known about the more recently discovered mTORC2 because there isn't a mTORC2-specific inhibitor (Thomson et al., 2009[113]). mTORC2 basal kinase activity is modulated by cellular ATP levels, in addition to its response to growth factors, amino acids, glucose, and hormones. This regulation ensures the integrity of the mTORC2 complex as well as the phosphorylation of Akt (Chen et al., 2013[19]). Akt is phosphorylated and made active when mTORC2 is activated. Akt then phosphorylates the transcription factors forkhead box O1 (FOXO1) and FOXO3 and restricts their activity (de Cavanagh et al., 2015[25]). When cells are under stress, FOXOs initiate processes that may stop cell growth and cause apoptosis as shown in Figure 4(Fig. 4). This entails activating the production of proteins that target the mitochondria, ligands for death receptors, and cyclin-dependent kinase inhibitors. Thus, the transcriptional activities of FOXOs are suppressed by Akt, which aids in promoting cell proliferation, growth, and survival. PKC-α (Protein kinase C- alpha), paxillin (a protein that controls actin filaments), and Rho GTPases are all activated by mTORC2, which affects the actin cytoskeleton's dynamic behavior and controls cellular morphology (Agarwal et al., 2013[2], Jacinto et al., 2004[56], Sarbassov et al., 2006[106]). Furthermore, mTORC2 activates the kinase serum- and glucocorticoid-induced protein kinase 1 (SGK1), which promotes the transport of ions and cellular growth (de Cavanagh et al., 2015[25]). Activated mTORC2 phosphorylates Akt, which then phosphorylates and inhibits TSC2, this process releases TSC2's restraining influence on mTORC1 or PRAS40. 

The interaction between mTORC1 and mTORC2 is complex, and when mTORC1 is turned on, RICTOR, a component of mTORC2's protein components phosphorylated which hinders mTORC2 signaling. Additionally, mTORC1 inhibits mTORC2 by insulin receptor substrate 1 (IRS1), phosphorylating growth factor receptor-bound protein 10 (GRB10), and alternatively Sin1 (Liu et al., 2014[78]). According to recent research, excessive PI3K signaling causes mTORC2 to interact with the ribosome, which activates mTORC2 (Zinzalla et al., 2011[129]). 

Initially, mTORC2 was first found to be involved in regulating the arrangement of the actin cytoskeleton (Foster and Fingar, 2010[38]). Further investigation has revealed that this complex also controls cellular survival, proliferation, and nutrient uptake (Zaytseva et al., 2012[124]).

### 2.2. Cellular Processes regulated by mTOR that promote aging

#### 2.2.1. Translation and Protein Synthesis

mTORC1 regulates crucial steps in protein synthesis, which affects gene expression and promotes cell survival and longevity. In addition to ribosome activity, efficient protein synthesis depends on the coordinated interaction of various components involved in initiating and extending the translation process (Chauvin et al., 2014[17], Thoreen et al., 2012[114]). The main targets of mTORC1 signaling are 4EBP1 and S6K, which control the start of translation. It has been shown that inhibiting mTORC-dependent translation increases lifespan and offers protection from an increased variety of age-related illnesses (Stallone et al., 2019[110]). Cap-dependent and cap-independent translation of mRNA molecules are regulated by mTORC1 via the phosphorylation of the translation inhibitors 4E-BP1 and 4E-BP2. The release of eIF4E is the result of this action. The initiation of translation for mRNAs that contain a 5' terminal oligopyrimidine tract (5' TOP) or a pyrimidine-rich translational element (PRTE) is significantly improved by mTORC1. Numerous proteins involved in translation, ribosomes, and metabolism are produced by many of such mRNAs (Hsieh et al., 2012[52]). Additionally, S6K1 and S6K2 are both known targets that are impacted by mTORC1-mediated phosphorylation. These kinases then activate ribosomal protein S6 through phosphorylation, aiding in the translation of proteins ((Saxton and Sabatini, 2017[107], Weichhart, 2018[120]). 

#### 2.2.2. Autophagy inhibition

**Figure 5:**
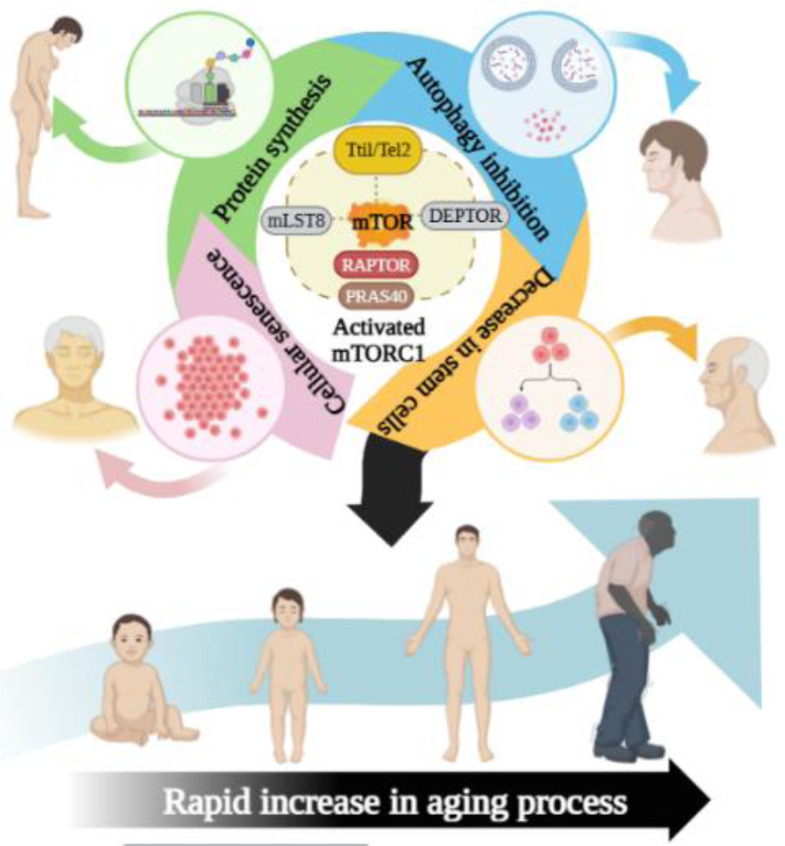
Figure 5. mTORC1 Role in Aging process: mTORC1 accelerates the aging process through various mechanisms, including increasing protein synthesis, inhibiting autophagy, depleting stem cell populations, and promoting cellular senescence.

Another distinct process under the control of mTORC1 is autophagy as shown in Figure 5(Fig. 5), which is crucial in promoting longevity. mTOR and autophagy are inversely related, meaning that when mTORC1 is active, autophagy is inhibited, and vice versa (Stallone et al., 2019[110]). The process of autophagy, which breaks down cytosolic components, is crucial during times of nutrient scarcity. This mechanism assists in the elimination of damaged organelles and offers a vital supply of substrates for the synthesis of energy in catabolic environments with low nutrient availability (Deretic, 2005[27]). mTORC1 regulates autophagosome formation and autophagic flux via the Ser/Thr kinase ULK1 (Deretic, 2005[27]). By phosphorylating ULK1, mTORC1 actively contributes to the inhibition of autophagy. As a result, inhibiting mTORC1 causes autophagy to start. It was once proposed that the eventual deterioration of autophagy with aging may be a factor in the accumulation of organelles and proteins that are damaged. It's still unclear what specifically caused this decline. By encouraging autophagy, especially mitophagy, which removes the aging-associated accumulation of dysfunctional mitochondria linked to aging and related conditions, Inhibiting mTORC1 has the possibility to delay the aging process (Saxton and Sabatini, 2017[107]).

#### 2.2.3. Cellular Senescence

Cellular senescence, which occurs after development and maturation and is characterized by decreased cellular functions, is influenced by mTORC1 signaling. Cells in this phase have a decreased capacity for replication without going through the apoptotic process (Blagosklonny, 2014[13]). According to some theories, Cellular senescence acts as a suppressor of tumors and promotes tissue restoration resulting from injury (Muñoz-Espín and Serrano, 2014[89]). However, Senescent cells might also directly contribute to aging (He and Sharpless, 2017[46]). Senescent cells release a variety of pro-inflammatory substances known as SASP (Senescence-Associated Secretory Phenotype) such as Tumor necrosis factor-α (TNF-α), Interleukin-6 (IL6), and Interleukin-1 (IL1) (Gimbrone Jr and García-Cardeña, 2016[42]). mTORC1 signaling regulate these pro-inflammatory cytokines (Conn and Qian, 2011[20]). This regulatory mechanism serves as crucial for the promotion of inflammaging, a distinct condition associated with aging that is characterized by a systemic elevation of pro-inflammatory agents (Stallone et al., 2019[110]).

#### 2.2.4. Stem Cell Function

mTORC1 has the potential to reduce the self-renewal and quiescence of hematopoietic stem cells, resulting in stem cell depletion and aging (Fernandes et al., 2021[35]). It has been found that as people age, their stem cell counts decline. Thus, inhibiting mTORC1 could be a viable strategy for preserving the stem cell reservoir, ensuring the ability to repair tissue injuries and slow the aging process (Chen et al., 2012[18]).

## 3. Hallmarks of Aging

Aging is typically signified by a steady decrease in overall physical health, which results in compromised functionalities and increased susceptibility to mortality, thereby limiting lifespan (Weichhart, 2018[120]). Recently, it has been proposed that aging is linked to several persistent traits. When there is no disease these traits whether taken individually or in combination are expected to more accurately indicate functioning capacities at an early age rather than chronological age (Baker 3rd, 1988[7]). Some of the aging hallmarks are known to be regulated by the mTOR network, as described below.

### 3.1. Mitochondrial dysfunction

Age-related health problems and the aging process may be influenced by genetic instability in mitochondrial DNA (mtDNA) (López-Otín et al., 2023[80]). It has long been accepted that a key factor causing aging and functional decline is mitochondrial dysfunction and it is a primary characteristic of aging cells (Kudryashova et al., 2020[70]). While performing oxidative phosphorylation, mitochondria consistently produce reactive oxygen species (ROS), which can be harmful to cells. Superoxide dismutase 2 (SOD2), a protective mechanism found in the inner mitochondrial matrix, scavenges these ROS. However, excessive ROS production has the potential to cause cellular damage, cellular senescence, and the aging process (Kanaki et al., 2016[65]). Multiple indicators of mitochondrial dysfunction are seen in aging cells such as ROS production is increased, DNA of mitochondria is mutated, electron transport and membrane potential are less functional, ATP synthesis is decreased, mitochondrial dynamics are disrupted, and mitophagy is unbalanced. Utilizing mechanisms that are involved in translation and transcription, mTORC1 controls the development, function, and evolution of mitochondria. It stimulates the translation of nuclear-encoded mRNA molecules linked to complex I and V components, mitochondrial ribosomal proteins, mitochondrial transcription factor A (MTFA), and fission process 1 (MTFP1) mRNA, among other mitochondria-associated molecules. As a result, mTORC1 activity boosts mitochondrial respiration and ATP generation, which leads to an increase in ROS production, which promotes aging. Through translational mechanisms, MTFP1 levels are decreased, increasing the hyperfusion of mitochondria while protecting cells from going through apoptosis. Studies have shown that mTORC1 controls the transcription of nuclear-encoded mitochondrial genes during prolonged stimulation via interacting with Peroxisome Proliferator-Activated Receptor Gamma Coactivator-1 (PGC-1) and the Yin-Yang 1 transcription factor (Papadopoli et al., 2019[94]). Mitophagy includes the specific destruction of mitochondria through autophagy, an excellent regulation system that ensures the recycling and destruction of damaged mitochondria (Kim et al., 2007[66]). mTORC1 plays a role in mitophagy regulation. The amount of mitophagy generated by a substance that uncouples mitochondria was reduced in cells lacking TSC2, where mTORC1 is overactive. Along with this, there was a slowdown in PARK2's migration to the outer mitochondrial membrane and a drop in PTEN-induced kinase 1 (PINK1) levels, which were regulated by mTORC1. These elements are believed to be important in mitophagy, which breaks down uncoupled mitochondria (Bartolomé et al., 2017[9]). These findings suggest that mTOR plays a part in a variety of mitochondrial activities, including their development, breakdown, and dynamic processes. Further research is needed to fully understand the degree to which mTOR contributes to mitochondrial dysfunction in aging cells.

### 3.2. Loss of proteostasis

Aging is linked with alteration in protein synthesis, degradation, and folding (Koga et al., 2011[69]). For example, the emergence of age-related diseases such as Parkinson's disease, Alzheimer's disease, and cataracts is influenced by the long-term presence and accumulation of proteins that are improperly folded or aggregated (Powers et al., 2009[97]). S6K and 4E-BPs are two important mTORC1 agents that have a major part in mTORC1-mediated protein synthesis activation. Additionally, Unc-51-like autophagy-activating kinase (ULK1) activity is partially restrained by mTORC1, which inhibits the autophagy process. It is becoming more and more clear that mTORC1 controls the equilibrium of protein synthesis and autophagy as a result of nutrient signals, keeping the cell's energy and protein levels stable (Lindqvist et al., 2018[75], Papadopoli et al., 2019[94]).

### 3.3. Cellular senescence

Senescent cells are distinguished by their inability to divide as a result of a persistent state of growth arrest. This arrest can occur as a result of telomere degradation over time (known as replicative senescence) or as a result of external stressors such as damage to DNA or abnormal activation of oncogenes (known as stress-induced premature senescence) (Matjusaitis et al., 2016[84]). Senescent cell accumulation over time is thought to possess an important effect on tissues' ability to regenerate themselves and is thought to be a factor in age-related problems like the depletion of stem cells, weakened immune response, the presence of chronic inflammation, and various persistent diseases (McHugh and Gil, 2018[85]). The distinct features of senescent cells are used as the foundation for their identification, possibly leading to their targeted identification or removal in the future via specific biomarkers. The reduced expression of LaminaB1 results in an enlarged size, flattened shape, and a disrupted nuclear envelope (Kudryashova et al., 2020[70]). It has been demonstrated that mTOR suppression stops stem cell aging and is essential for encouraging the release of chemicals known as secretory phenotypes, which are molecules that identify senescent cells. Rapamycin, for instance, has been found to reduce the secretion of the IL-1R-dependent senescence-associated secretory phenotype by preventing the translation of IL-1 mRNA. As a result of this effect, the expression of inflammatory genes controlled by the pro-inflammatory transcription factor nuclear factor-B (NF-B) is reduced. Additionally, it's thought that mTORC1 and MAPK (Mitogen-activated protein kinase) interact to improve MK2 kinase translation. This interaction prevents a protein called ZFP36 ring finger protein-like 1 (ZFP36L1) from degrading several SASP factor transcripts (Papadopoli et al., 2019[94]). Ultimately, mTORC1 signaling's importance in aging is probably due to its exceptional ability to regulate a variety of crucial cellular processes as discussed above.

## 4. Impact of mTOR in Diseases associated with Aging

### 4.1. Cancer

In human cancers, mTOR is frequently activated. Research suggests that the improper growth of cells and their metabolic regulation substantially contribute to the occurrence and spread of cancer (Cornu et al., 2013[21]). Cancer is a condition associated with aging, and treatments that slow down aging may be able to delay the development of cancer. It makes sense to assume that rapamycin's potential to slow down aging will eventually result in a delay in the development of cancer (Blagosklonny, 2013[14]). According to a widely accepted theory, cancers that rely on the triggering of the oncoprotein AKT require later stimulation of mTORC1 to fuel their development into tumors. This propensity for mTORC1 signaling in some cancers renewed interest in developing the mTORC1 inhibitor rapamycin as a cancer treatment (Guertin and Sabatini, 2007[44]).

### 4.2. Metabolic disorders

Metabolic disorders are conditions that disrupt normal metabolic processes, potentially causing problems with energy production, nutrient utilization, and hormone regulation. Type 2 diabetes and obesity are notable examples. mTOR signaling disruption has been linked to metabolic irregularities, and inhibiting mTOR activity holds promise in restoring metabolic equilibrium and improving outcomes in these disorders. For anabolic metabolism, utilization, storage of energy, and normal cell and tissue development, there must be intermittent, temporary activation of mTORC1 signaling within a physiologically acceptable quantity of nutrient availability. On the other hand, ongoing mTORC1 signaling activation occurs in conditions involving excessive nutrient intake or persistently elevated levels of nutrients like glucose and amino acids (Um et al., 2004[116]). Certainly, research shows that mTORC1 activity is elevated in animal models affected by obesity and metabolic disorders, whether due to hereditary factors or induced by dietary conditions. The heart, skeletal muscles, adipose tissue, vasculature, liver, and other metabolically active organs and tissues exhibit this elevated activity. Insulin resistance and hyperinsulinemia are additional symptoms of metabolic syndrome and obesity (Yang and Ming, 2012[123]).

### 4.3. Neurodegenerative disorders

The importance of mTOR communication in maintaining protein equilibrium, which includes both synthesis and autophagic breakdown, holds particular significance within the brain (Hoeffer and Klann, 2010[48]). mTORC1 improves synapses through protein synthesis-mediated strengthening, which aids in learning abilities and the formation of memories.

The influence of mTOR signaling on autophagy extends to a variety of neurodegenerative disorders. An aberrant build-up of misfolded proteins is a hallmark of neurodegenerative diseases like Parkinson's, Huntington's, and Alzheimer's, disease. This accumulation causes neurons to die and subsequently manifests as dementia, involuntary shaking, and problems with memory and language (Dazert and Hall, 2011[24]). The administration of rapamycin prompted the autophagic elimination of harmful misfolded proteins, resulting in a decrease in pathological manifestations in murine animals of Huntington's and Alzheimer's diseases (Ravikumar et al., 2004[100]). 

### 4.4. Cardiovascular diseases

**Figure 6:**
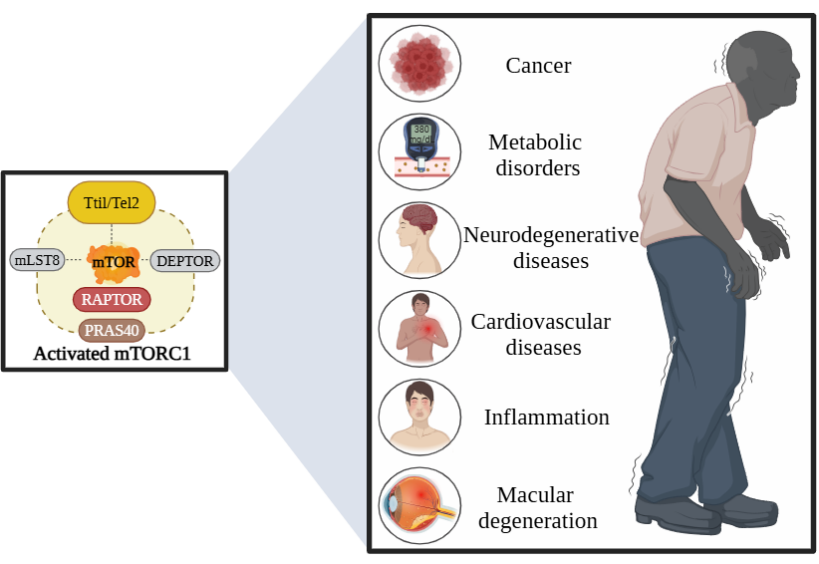
Figure 6. Aging-related diseases caused by hyperactivation of mTORC1

Cardiovascular disorders encompass many different kinds of heart as well as blood vessel illnesses such as heart failure, coronary artery disease, and a stroke. The role of mTOR1 signaling in these diseases is attributed to a variety of interactions that link metabolic signaling with immune and inflammatory responses. As a result, altering mTOR1 signaling offers a promising strategy for treating cardiovascular issues as shown in Figure 6(Fig. 6). Furthermore, prolonged Akt activation has frequently been linked to cardiac dysfunction (Jia et al., 2014[57], Xu et al., 2013[122]), as well as prolonged mTOR1 inhibition may increase PI3K and Akt signaling (Lamming et al., 2013[71]). As a result, it appears that targeting mTOR1 manipulation will be more beneficial in improving the management of cardiovascular diseases (Yang and Ming, 2012[123]).

### 4.5. Inflammation

mTOR is crucial for assessing the fact that a T cell gets stimulated or remains unresponsive to antigens (Powell and Delgoffe, 2010[96]). When mTOR is lacking, mice's CD4+ T cells do not develop into active effector T cells but rather become regulatory T cells with diminished metabolic activity (Delgoffe et al., 2009[26]). Autoimmune conditions such as systemic lupus erythematosus (SLE) are associated with elevated mTOR signaling. Additionally, inhibiting the mTOR pathway appears to be an intriguing therapy for the treatment of autoimmune diseases, to increase regulatory T cells, and to limit autoimmune damage (Fernandez and Perl, 2010[36]).

### 4.6. Macular Degeneration

In Western countries, macular degeneration associated with aging is the leading cause of blindness (Leung and Landa, 2013[74]). Macular degeneration is linked to mTORC1, which influences the retinal pigment epithelium and photoreceptors. Its activation promotes drusen formation and increases the production of pro-inflammatory molecules, such as SASP. This inflammation causes damage to the RPE and photoreceptors, which are key cells in macular degeneration (Huang et al., 2019[54]). Age-related macular degeneration in rats has demonstrated that rapamycin reduces the incidence and severity of retinopathy. Rapamycin appeared to reduce the need for intravitreal injections of anti-VEGF medications in human patients by about 50% (Nussenblatt et al., 2010[91]).

## 5. Utilizing mTOR Inhibitors as Anti-Aging Interventions

### 5.1. Rapamycin and Rapalogs

A naturally occurring substance called rapamycin was discovered in the soil which contains the bacterium Streptomyces hydroscopius and it was initially employed as an immunosuppressant and antifungal (Alvarado et al., 2011[3]). The discovery of Rapamycin's specific targeting of mTOR, a key regulator in cellular processes, accelerated research into its anti-aging properties. Challenges with rapamycin's solubility and how it behaves in the body prompted initiatives aimed at improving these properties. This work resulted in the creation of the first batch of rapalogs, also known as rapamycin analogs. These artificial substances have similar modes of action and are derived from rapamycin. Their construction aims to improve the pharmacological properties of rapamycin by increasing stability and the degree of their absorption by the body (Zaytseva et al., 2012[124]). It is impossible for mTOR to bind to RAPTOR when rapamycin and FKBP12 bind to the FRB. It is believed that when mTORC1 is separated from its main targets like 4E-BPs and S6Ks, its activity decreases (Oshiro et al., 2004[92]). Unlike mTORC1, mTORC2 doesn't interact with rapamycin/FKBP12, which is thought to be the cause of mTORC2's resistance to rapamycin's immediate effects (Sarbassov et al., 2006[106]). Rapamycin and its analogs may be effective anti-aging medications because of their capacity to inhibit mTORC1 and thereby hinder both the proliferation and growth of cells (Faivre et al., 2006[33]). Rapalogs, also known as rapamycin analogs have better pharmacokinetic qualities, and the same mechanism of action like rapamycin. Rapalogs retain their ability to interact with mTOR and FKBP12, resulting in a rapamycin-like mode of action (Liu et al., 2009[79]). While rapalogs are generally less effective at targeting mTORC2, long-term drug exposure may disrupt mTORC2 structure thereby suppressing Akt signaling (Zeng et al., 2007[125]). The rapamycin-FKBP12 complex gradually sequesters the internal mTOR pool, inhibiting its participation in mTORC2 assembly, and as a result, indirectly inhibits mTORC2 (Zoncu et al., 2011[130]). 

Temsirolimus, a rapamycin dihydroxy methyl propionic acid ester, was purposefully created to enhance rapamycin the ability to dissolve. This modification allows it to be administered both orally and intravenously (Dudkin et al., 2001[30]). Temsirolimus inhibits mTORC1 by binding to FKBP-12, an intracellular protein that forms a complex with mTOR. This complex prevents mTOR from interacting with its substrates and partners, thereby inhibiting its signaling (Klümpen et al., 2010[68]).Thus, it prevents the mTOR-driven phosphorylation of 4E-BP1 and S6K1 by inhibiting mTOR activity. This inhibition reduces the expression of essential proteins that control the cell cycle (Rini, 2008[101]).

Everolimus is a rapamycin analog that can be taken orally. Its creation was intended to increase rapamycin's oral bioavailability. Everolimus has better pharmacokinetic characteristics than rapamycin, such as a shorter half-life (28 hours as opposed to 60 hours) and slightly higher absorption rate (Augustine and Hricik, 2004[5]). Everolimus, like other rapalogs, form complexes with the intracellular protein FKBP12. The Everolimus-FKBP12 complex binds to mTORC1's FRB domain, inhibiting kinase activity. Everolimus effectively inhibits mTORC1, reducing the phosphorylation of downstream targets such as p70S6 kinase and 4E-BP1, which suppresses protein synthesis. These molecular actions are believed to contribute to cellular rejuvenation and the suppression of aging-related mechanisms (Klümpen et al., 2010[68]).

Deforolimus, a phosphorus-containing rapamycin analog, was developed using insights from computational modeling analyses. Deforolimus has better pharmacological and pharmaceutical properties than rapamycin, including improved solubility in water, improved stability of chemicals, and increased absorption rate (Mita et al., 2008[87]). Deforolimus, also called ridaforolimus, can inhibit the mTORC1. It leads to decreased anabolism and increased catabolism, which may have anti-aging effects (Blagosklonny, 2017[12]).

### 5.2. Plant bioactive compounds influencing mTOR

#### 5.2.1. EGCG (Epigallocatechin Gallate)

EGCG (Epigallocatechin Gallate), the extensively researched polyphenolic component found in green tea, has potent antioxidant properties and is thought to hold therapeutic promise for a variety of conditions. Keloid fibroblasts and HMC-1 cells co-cultured with EGCG and it was found that phosphorylation of Akt, S6K, and 4E-BP1 was decreased in a dose-dependent manner (Zhang et al., 2006[126]). EGCG activated AMPK, suppressing downstream targets like 4E-BP1 and mTOR, resulting in a reduction in the translation of mRNA in human hepatoma cells with both positive and negative p53 status (Huang et al., 2009[53]). AMPK directly phosphorylates and activates TSC2, and thus inhibits mTORC1. Furthermore, it inhibits Raptor, a subunit of mTORC1 necessary for its activity (Garza-Lombó et al., 2018[40]). Due to a decrease in mRNA translation, there is less protein synthesis which can lead to anti-aging intervention.

#### 5.2.2. Resveratrol

Red wine and grapes contain the polyphenolic flavonoid (Resveratrol) which has antioxidant, anti-inflammatory, neuroprotective, and anticancer properties (Marques et al., 2009[82]). Resveratrol inhibited the mTOR signaling pathway in human U251 glioma cells, and when combined with rapamycin, it increased the cell mortality caused due to resveratrol (Jiang et al., 2009[58]). Oxidized LDL-induced proatherogenic mTOR pathway was inhibited by resveratrol. in smooth muscle cells (SMCs), which significantly decreased DNA synthesis and smooth muscle cell proliferation (Brito et al., 2009[15]). The ability of resveratrol to activate AMPK in both Estrogen receptor-positive and negative breast cancer cells has recently come to light. This activation inhibits mTOR, as well as 4E-BP1 signaling and mRNA translation, which are all downstream effects of mTOR. Furthermore, it has been demonstrated that resveratrol induces Sirtuin type 1 (SIRT1) expression, which is the basis for AMPK activation. 

#### 5.2.3. Curcumin

According to an increasing amount of research, curcumin may inhibit mTOR signaling in order to exert its antiproliferative effects, which could lead to the development of a novel class of mTOR inhibitors. A naturally occurring polyphenolic substance called curcumin is derived from the rhizome of the Curcuma longa plant (Goel et al., 2008[43]). Curcumin was found to inhibit rhabdomyosarcoma cells' basal or IGF-I-triggered mobility, stimulate programmed cell death, and reduce the growth of cells (Beevers et al., 2009[10]). In human melanoma cells, curcumin induces autophagy by suppressing the mTOR signaling pathway (Bahrami et al., 2021[6]). Curcumin obstructed the phosphorylation of mTOR and its targets like S6K1 and 4E-BP1. This implies that it has the potential to inhibit the mTOR activity which ultimately reduces the aging process.

#### 5.2.4. Quercetin

Quercetin is a beneficial bioflavonoid that blocks mTOR action in a variety of ways through various pathways, making it an effective option for treating age-related diseases and other conditions associated with mTOR dysregulation (Bruning, 2013[16]). Quercetin can activate AMPK which inhibits mTORC1, resulting in decreased protein synthesis and cell growth. This AMPK-mediated suppression of mTORC1 is thought to be one mechanism by which quercetin may exert anti-aging effects, promoting cellular balance and reducing aging processes (Cui et al., 2022[22]).

#### 5.2.5. Berberine

In MHCC97-L and HepG2 cell lines, berberine has been found to activate Beclin-1, resulting in cell death through autophagy. Berberine simultaneously interferes with the mTOR signaling by decreasing Akt functions and enhancing P38 MAPK signaling. All of these mechanisms work together to allow berberine to block the mTOR pathway, which in turn affects cell growth, survival, and other cellular functions (Wang et al., 2010[118]).

#### 5.2.6. Caffeine

It has been demonstrated that caffeine activates AMPK, a protein that negatively regulates mTOR. There is evidence that caffeine inhibits Akt signaling., which is a positive regulator of mTOR. Caffeine can inhibit certain phosphodiesterase, which can affect cyclic adenosine monophosphate (cAMP) levels it is a signaling molecule that can affect mTOR activity (Saiki et al., 2011[104]). All of these mechanisms work together to allow caffeine to block the mTOR action.

### 5.3. mTOR kinase inhibitors that compete with ATP

Inhibitors that target mTOR kinase activity by competing with ATP are a newer class of mTOR inhibitors. It was developed as a result of the clinical limitations of rapamycin-based therapies. These blockers work by specifically inhibiting the catalytic activity of mTOR domains. These drugs have a key mechanistic benefit because they can block mTORC1 and mTORC2 signaling as well as prevent the PI3K signaling pathway (Dowling et al., 2010[29]). There have been many kinase inhibitors developed, including XL-388, INK-128, PP30, PP242, WYE-354, WAY-600, WYE-687, OSI-027, AZD-8055, and WyethBMCL-200908069-2. Similar to rapalogs, mTOR kinase inhibitors that compete with ATP have demonstrated the capacity to obstruct angiogenesis, inhibit cell cycle progression, and decrease protein translation in various types of cancer cell lines (Mamane et al., 2006[81]). In fact, compared to rapalogs, these inhibitors have demonstrated even more potent inhibition of cell growth and proliferation. In contrast to the limited impact of rapamycin treatment, Tyrosine Kinase Inhibitors (TKIs) such as WYE-354 WAY-600, and WYE-687 significantly reduced overall protein synthesis in breast cancer cells by nearly 50% (Bärlund et al., 2000[8]).

### 5.4. Inhibitors that inhibit both mTOR and PI3K

This category includes GNE477, NVP-BEZ235, PI-103, XL765, and WJD008. These inhibitors target both mTOR and PI3K at the same time. These target ATP binding sites with comparable efficiency, which prevents them from selectively inhibiting mTOR-specific functions (Zhou et al., 2010[128]). They are capable of affecting three important enzymes in the PI3K signaling pathway that is Akt, mTOR, and PI3K. In a study comparing NVP-BEZ235's effects with those of everolimus on a variety of cancer cell lines with various genetic backgrounds and mutational profiles, NVP-BEZ235 showed superior efficiency in preventing cell proliferation (Serra et al., 2008[108]).

## 6. Safety and Tolerability Considerations of mTOR

### 6.1. Side effects

Unfavorable outcomes that may result from treatment or intervention are known as side effects. Inhibiting mTOR has the potential to have a number of adverse effects because it plays a crucial role in controlling development, normal growth, and the balance and function of organs (Zhang et al., 2019[127]). Everolimus and temsirolimus, two more recent additions to the rapalog family, share several distinct side effects. It's interesting to note that as drug dosage increases, some side effects, like pneumonitis or mucocutaneous effects, seem to become more common. On the other hand, many of these side effects are unique and unpredictable (Pallet and Legendre, 2013[93]). Table 1(Tab. 1) (References in Table 1: Feng et al., 2000[34]; Franz et al., 2013[39]; Juss et al., 2012[63]; Nguyen et al., 2019[90]; Pallet and Legendre, 2013[93]; Qiao et al., 2002[98]; Stokman et al., 2002[111]; Tremblay and Marette, 2001[115]; Zhang et al., 2019[127]) contains an in-depth overview of the consequences of mTOR inhibitors. 

## Conclusion

The mTOR signaling is being recognized as a key participant in the complexities of aging, controlling cellular activities that collectively regulate longevity and health span. This extensive study has clarified the various functions of mTOR inhibitors in relation to aging, highlighting their efficacy as therapeutic approaches to counteract age-related decline and illnesses. The impact of mTOR inhibition has been evident from the molecular to the clinical sphere. mTOR acts as a monitor at the molecular level, collecting a variety of information ranging from nutrition status to the availability of energy and regulating cellular responses that govern development, metabolic processes, and controlling stress. The improper regulation of mTOR has thrown a spotlight on aging, tying its disruption to a variety of age-related illnesses that affect our aging process. Research into the impact of mTOR inhibitors, particularly rapamycin, and rapalogs, has revealed their amazing capacity to rebalance cellular processes. Autophagy, an essential part of cell quality control, is regulated by mTOR, and blocking this pathway has been demonstrated to stimulate autophagic processes, boosting cellular resiliency and postponing senescence. Further highlighting its potential to slow the effects of aging, mTOR inhibition also appears to benefit mitochondrial function, the foundation of energy metabolism, and the control of oxidative stress. Perhaps most effectively capturing the attraction of mTOR inhibitors is their effect on health span improvement and lifespan extension. Preclinical studies in research animals have shown that mTOR inhibitors can increase longevity, introduce youthfulness into aging tissues, and inhibit the development of age-related illnesses. Careful calibration is required in the dose schedules of mTOR inhibitors to maintain a balance between the risk of side effects and the increase in lifetime. Furthermore, given the importance of preserving physiological homeostasis in various organ systems, the tissue-specific consequences of mTOR inhibition need further investigation. T fully utilize mTOR inhibitors as clinical therapies, it will be essential to overcome these issues as research develops. The potential of mTOR inhibition has primarily improved aging research. Its many-faceted impacts on cellular functions, longevity, and age-related disorders come together to provide a peek into a future in which the process of aging may be modified. As the knowledge of mTOR signaling develops, its careful modulation has the ability to bring in a period in which not just the number of years lived increased, but the overall value of those years is significantly improved. mTOR inhibitors appear as torch bearers, revealing a route loaded with potential on the path to a future when age-related diseases might be managed. As scientists explore further into mTOR signaling, the promise of mTOR inhibitors and the rapidly advancing frontiers of scientific knowledge are propelling us into an exciting new period where the goal of healthier, younger aging assumes a central role.

## Notes

Kamil Kuca and Rachna Verma (School of Biological and Environmental Sciences, Shoolini University of Biotechnology and Management Sciences, Solan 173229, India; E-mail: rachnac83@gmail.com) contributed equally as corresponding author.

## Declaration

### Competing interests 

The authors declare no competing interests. They have no financial relationship with the organization that sponsored the research.

### Author's contribution

Komal Raghuvanshi: Investigation, Writing-original draft & Formal analysis; Disha Raghuvanshi: Visualization; Dinesh Kumar: Formal analysis; Eugenie Nepovimova: Formal analysis & Data curation; Marian Valko: Resources, Investigation & Conceptualization; Kamil Kuca: Resources, Investigation & Conceptualization; Rachna Verma: Supervision, Investigation & Conceptualization.

### Funding

This work was supported ba the project - CZ.10.03.01/00/22_003/0000048, MH CZ - DRO (UHHK, 00179906), Project Excelence FIM UHK 2203 and Project Excelence PrF UHK 2207/2025-2026, and GA23-05857S. 

### Usage of Artifical intelligence

We declare that AI was not use for the preparation of this manuscript.

## Figures and Tables

**Table 1:**
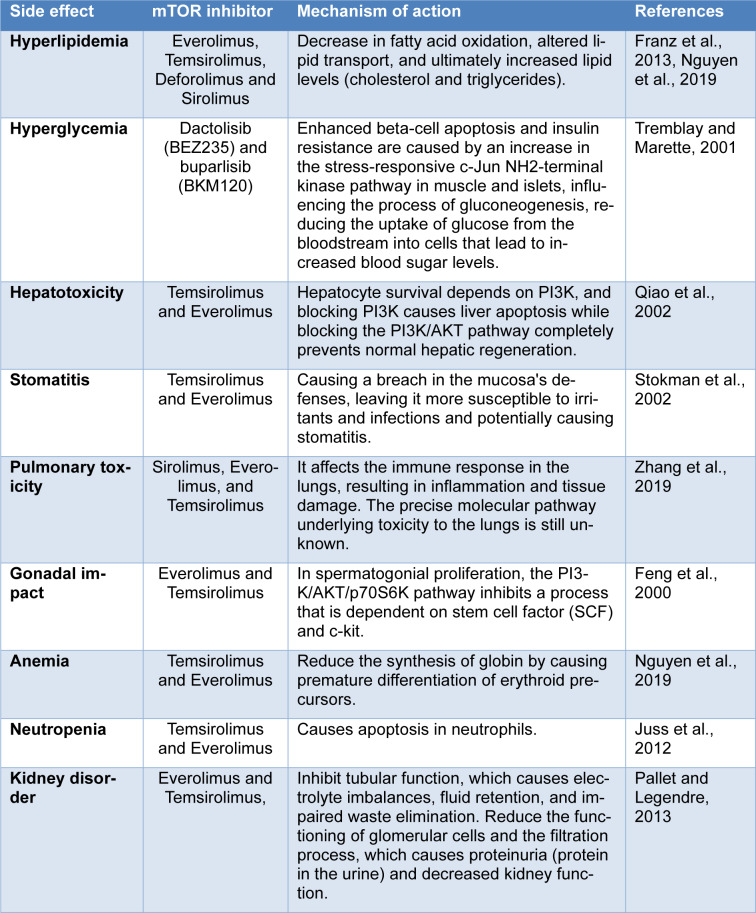
Table 1. Adverse effects of mTOR inhibitors
